# Utilizing wearable technology to increase physical activity in future physicians: A randomized trial

**DOI:** 10.1016/j.pmedr.2018.09.004

**Published:** 2018-09-11

**Authors:** Joanne DiFrancisco-Donoghue, Min-Kyung Jung, Alexander Stangle, William G. Werner, Hallie Zwibel, Patricia Happel, Jerry Balentine

**Affiliations:** a Department of Osteopathic Medicine, New York Institute of Technology College of Osteopathic Medicine (NYIT-COM) Old Westbury, NY, USA; b Office of Research, NYIT-COM, Old Westbury, NY, USA; c NYIT-COM, Old Westbury, NY, USA; d Department of Family Medicine, NYIT-COM, Old Westbury, NY, USA; e Department of Physical Therapy, NYIT, Old Westbury, NY, USA; f Center for Sports Medicine NYIT-COM, Old Westbury, NY, USA; g Office of the Vice President NYIT-COM, Old Westbury, NY, USA

**Keywords:** Fitbit, Exercise, Medicine, Obesity, Body composition

## Abstract

This study examined the use of activity trackers alone or combined with weekly communication through email to improve activity and body composition over one academic year in medical students. This randomized clinical trial conducted at the New York Institute of Technology from July 7, 2016 through June 4, 2017 enrolled 120 medical students. The first group (Fitbit-Plus) wore activity trackers and received weekly emails offering fitness challenges and lifestyle modification challenges. The second group (Fitbit-Only) received only activity trackers and did not receive weekly emails. The third group (Control) was asked not to purchase an activity tracker of any kind throughout the study. All groups had a body composition analysis prior to the start of the academic year and at the end of the first academic year. Outcome measures included step count and body composition (body fat percentage and lean body mass). The results showed the overall mean daily steps were greater in the Fitbit-Plus group than the Fitbit-Only group for the academic year (7429 ± 2833 vs. 6483 ± 2359) with only months April and May showing a significant difference between the groups (p = 0.011; p = 0.044). Body fat percentage decreased in the Fitbit-Plus overweight women (2.1 ± 1.6%) lean body mass increased in the Fitbit-Plus group in overweight men (2.4 ± 4.6 lbs.). A subsequent finding of this study showed improved body composition in a small sub-group of over-weight students. Weekly behavioral challenges combined with an activity tracker increased step count in medical students compared to an activity tracker alone.

Clinicaltrials.gov Identifier: NCT02778009.

## Highlights

• Activity trackers in conjunction with behavioral challenges yielded an increase in step count compared to wearing an activity tracker alone.
• Incorporating lifestyle interventions while utilizing activity trackers improved body composition in overweight medical students.
• Activity trackers with lifestyle interventions may influence future physicians to provide recommendations on public health guidelines.

## 1 Introduction

Physical inactivity is the fourth leading risk factor for global mortality resulting in an estimated 3.2 million deaths per year (Dacey et al., 2014). “Healthy People 2020” has called for an increase in the number of physician office visits that include counseling or education related to physical activity to help combat this global epidemic (Dacey et al., 2014). The American College of Sports Medicine (ACSM) has also implemented a program called, “*Exercise is Medicine*®”, to encourage physicians to assess a patient's activity level at every visit. Despite these encouraging programs, there has been a decline in the amount of education regarding the benefits of physical activity and health behavior guidelines provided to medical students (Wolf and Scurria, 1995; Garry et al., 2002; Cardinal et al., 2015). There are currently no recommendations by the Association of American Medical Colleges (AAMC) to incorporate nutrition or wellness education into the medical school curriculum (Cardinal et al., 2015; Adams et al., 2010). It has been reported that physical activity habits of medical students influence their counseling practices after graduation and that a physician's lifestyle can influence the behavior of their patients (Dacey et al., 2014; Lobelo et al., 2009; Abramson et al., 2000). A study conducted by Stephens et al. determined that physical fitness levels declined during medical school, most notably during the preclinical years (Stephens et al., 2012). Although current literature supports the notion that medical schools should incorporate programs and practical requirements to increase physical activity and wellness education to physicians, how to incorporate these programs is an area of question (Frank et al., 2008; Institute of Medicine, n.d.).

Over 10% of adults now wear an activity tracker and the ACSM consensus states activity trackers were the hottest trend for 2017 (Thompson, 2016). These wearable devices are widely used to track and promote physical activity by employers who offer incentives for activity, insurance companies and researchers to help monitor fitness. However, the long term effectiveness to influence lifestyle changes and physiological outcomes has been mixed (Jakicic et al., 2016; Finkelstein et al., 2016). Integrating wearable technology and wellness education into medical education may provide opportunities for these future practitioners to stay abreast of scientific advances while encouraging healthy habits and maintenance of healthy behaviors.

The purpose of this randomized trial was to: 1) determine if implementation of a program designed to increase physical activity and educate medical students is feasible (drop-out rate); 2) examine the effects of utilizing activity trackers alone or combined with weekly emails of encouragement and challenges to improve activity measured by step count throughout the first year of medical school and; 3) examine if this program could affect body composition.

## 2 Methods

### 2.1 Study oversight

FIT-Physician was a randomized clinical trial conducted at the New York Institute of Technology College of Osteopathic Medicine (NYIT-COM), Old Westbury, New York from July 7, 2016 through June 4, 2017. This study was approved by the NYIT Internal Review Board and was registered on Clinicaltrials.gov Identifier: NCT02778009. A total of 120 first year medical students enrolled at NYIT-COM provided written informed consent. All student data were coded and kept confidential. Only the authors had access to the data associated with this study.

### 2.2 Participants

Recruitment was conducted via social media and email prior to the start of the academic medical school year at NYIT-COM. Eligibility was assessed by a self-reported web based questionnaire beginning in July 2016. Eligibility criteria included: 1) between 17 and 50 years of age and; 2) incoming 1st year NYIT-COM medical student. Exclusion criteria included: 1) pregnancy or; 2) current use of an activity tracker.

### 2.3 Study outcomes

The primary outcome was the difference in daily step counts between groups. The secondary outcomes included changes in body composition (body fat percentage, lean body mass, BMI, weight) and self-reported physical activity. The control group was instructed not to wear activity trackers in order to act as controls for the group that wore activity trackers.

### 2.4 Intervention

**Fig. 1:**
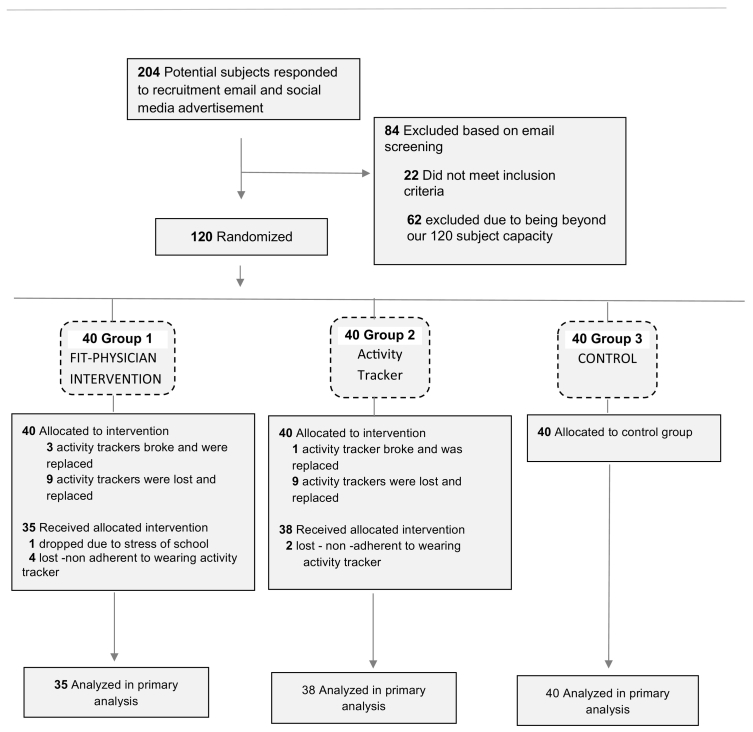
Study flow chart.

In this 10 month study, 120 first year medical students (age 22.9 ± 2.9) were randomly assigned, with stratification by sex and age, to one of three groups (Fig. 1. Study flow chart).

The first group (Fitbit-Plus) (Fitbit Flex™, Fitbit Inc. San Francisco, CA) (Imboden et al., 2017) wore wrist activity trackers and were encouraged to participate in mentored weekly walk/runs that were conducted by faculty and administration of the medical school. Weekly emails were sent to this group offering fitness challenges in an attempt to foster an increase in step count. These emails were sent every Sunday for 39 weeks (the entire length of the first year of medical school). Throughout the study the men and women in the Fitbit-Plus group were routinely encouraged to compete with each other in weekly challenges. These weekly emails provided feedback on group step count as a total compared with the other group (Fitbit-Only). Additionally, the Fitbit-Plus group was given feedback on step count separated by men and women. Fitness challenges were based on suggestions from the Fitbit website and by the underlying goal to increase activity. These emails included reminders to encourage and increase step count by 500 steps daily per week. The goal was to attain at least 10,000 steps daily which is equivalent to approximately 30–45 min of walking which is the recommendation of the Surgeon General (Fitbit, n.d.; Executive Summary, n.d.; Duncan et al., 2018). This was reinforced every week in the emails with the group's current activity status. Participants were encouraged to park their cars as far as possible to add extra steps, and were urged to take the stairs in lieu of the elevators throughout the day.

The second group (Fitbit-Only) received Fitbits only and received no emails throughout the study. This group was given instructions on how to use/sync the Fitbit but was not given any goals.

The third group (Control) was asked not to purchase an activity tracker of any kind throughout the duration of the study.

### 2.5 Outcome measures

#### 2.5.1 Activity

Daily step count was measured for 39 weeks using a Fitbit Flex™ wrist worn activity tracker for the Fitbit-Plus group and the Fitbit-Only group. This particular model was chosen due to its small size and ease of use. The participants were instructed on how to create an account with their personal information. Each participant was emailed a link to connect their Fitbit to a web based dashboard designed for collecting mass data from this particular Fitbit (Fitabase, ™ 2015 Small Steps Labs LLC) The Fitabase™ dashboard was customized for this study. This particular dashboard allowed each tracker to be monitored individually. The dashboard monitored the battery life of each Fitbit, when participants connected their Fitbit, and how often they synced their device to the dashboard. These devices were able to store data for up to 30 days, however, if more than two weeks passed without a participant syncing their device, a reminder email was sent to them.

#### 2.5.2 Body composition

Fat mass, body fat percentage, weight, lean body mass, and body mass index (BMI) were assessed using dual-energy x-ray absorptiometry (Lunar™ I-Dexa General Electric, Atlanta, GA). Any woman who was possibly pregnant was excluded due to the radiation by the device.

All groups had body composition analyzed in August prior to the start of the academic year and then had a body composition scan at the end of the first academic year.

#### 2.5.3 Survey/self-reported questionnaire

Prior to the study and upon completion of the study the participants were asked if they exercised and the number of days they exercised currently. The participants' opinion on Fitbits was anonymously assessed after completion of the study using a self-made survey through Survey Monkey™ with three questions. Question 1; do you think this study will encourage you to incorporate physical activity counseling into your practice when you become a practicing physician? Question 2; did wearing an activity tracker help you to become more aware of your activity for 10 months? Question 3; as a future physician would you recommend using an activity tracker for your patients? These questions were answered using a Likert scale originally of five categories (Strongly Agree, Agree, Neutral, Disagree, Strongly Disagree), which were collapsed for the analysis purpose of capturing trends in the data with three categories of Agree, Neutral, and Disagree.

#### 2.5.4 Statistical methods

An a priori power analysis estimated that a sample size of 40 participants per group (120 participants overall) would provide 80% power to detect 1000 steps difference between groups assuming the mean steps of 6000 (±1500) for US adults of ages 18–29. Generalized Estimating Equation (GEE) was used to fit a model to the longitudinal data of daily steps and test the significance of the difference between the Fitbit Plus and Fitbit Only groups across the nine months of the study. The mean and the standard deviation were computed and analysis of variance was employed to describe the trends by the groups in the difference of the body composition measures and self-reported physical activity. Chi-square tests were conducted to check the significant difference of the proportions for all survey data. Statistical significance was determined with p-value <0.05 after multiplicity correction using a Benjamini-Hochberg procedure.

## 3 Results

One hundred and twenty participants were randomized for this study. One hundred and thirteen participants (94%) were analyzed in the final primary data. One participant discontinued the study due to stress of medical school and six participants were not compliant wearing the activity tracker (if they did not sync the tracker for over 1 month). It is worth noting that 18 (23%) of the activity Fitbits were lost throughout the study and were replaced by the institution conducting the study. Four activity trackers malfunctioned and were replaced by the manufacturer (see Fig. 1).

Baseline measurements were significantly different between men and women therefore baseline measurements were described separately by gender (Table 1. Characteristics of body composition measures by group pre and post intervention).

**Table 1:** Characteristics of body composition measures by group pre and post intervention.

Gender- men	Pre				Post			
	Fitbit Plus group 1 (n = 15)	Fitbit Only group 2 (n = 19)	Control group 3 (n = 19)	p-Value⁎	Fitbit Plus group 1 (n = 15)	Fitbit Only group 2 (n = 19)	Control group 3 (n = 19)	p-Value
Weight (kg)	78.2 (12.6)	79.4 (16.8)	82.5 (9.3)	0.97	79.5 (29.3)	79.5 (13.3)	81.1 (12.5)	0.94
Body fat %	22.2 (6.6)	24.3 (7.7)	24.2 (7.2)	0.97	22.8 (6.8)	24.5 (6.3)	24.4 (6.8)	0.94
Body mass index (BMI)	25.3 (3.2)	25.7 (4.8)	25.9 (3.2)	0.97	25.4 (3.2)	25.8 (4.3)	25.3 (3.7)	0.94
Lean body mass (kg)	57.4 (8.5)	56.2 (7.9)	59 (6.4)	0.97	57.9 (8.4)	56.7 (7.2)	55.7 (10.7)	0.94
Fat mass (kg)	17.7 (6.9)	20.2 (10.2)	19.5 (7.9)	0.97	18.5 (7.8)	20.2 (8.2)	20.2 (7.8)	0.94
Days exercised	4.1 (1.9)	3.9 (1.5)	4.0 (1.6)	0.97	3.7 (1.9)	2.7 (1.7)	3.6 (2.2)	0.94


Gender- women	Pre				Post			
	Fitbit Plus group 1 (n = 20)	Fitbit Only group 2 (n = 19)	Control group 3 (n = 21)	p-Value	Fitbit Plus group 1 (n = 20)	Fitbit Only group 2 (n = 19)	Control group 3 (n = 21)	p-Value
Weight (kg)	58 (10.4)	60.5 (11.8)	56.2 (7.8)	0.96	57.7 (9.8)	60.8 (11.8)	59.2 (11.6)	0.74
Body fat %	30.6 (7.0)	34.9 (6.9)	32.0 (5.6)	0.78	29.2 (6.9)	34.4 (7.2)	31.4 (5.7)	0.39
Body mass index (BMI)	21.6 (2.7)	22.5 (3.6)	21.8 (2.7)	0.96	21.4 (2.9)	22.7 (3.5)	22.4 (3.4)	0.68
Lean body mass (kg)	37.7 (6.4)	36.6 (5.8)	35.8 (4.7)	0.96	38.5 (6.0)	37.0 (5.6)	38.1 (6.3)	0.74
Fat mass (kg)	18 (6.8)	21.7 (8.2)	19.1 (6.7)	0.81	17.1 (6.6)	21.2 (7.8)	18.9 (6.4)	0.58
Days exercised	3.5 (1.6)	3.7 (1.8)	3.6 (1.9)	0.96	3.7 (1.9)	2.8 (1.8)	3.0 (1.8)	0.58

⁎ p-Values were adjusted using Benjamini-Hochberg procedure to control familywise error rate per session in each gender group.

**Fig. 2:**
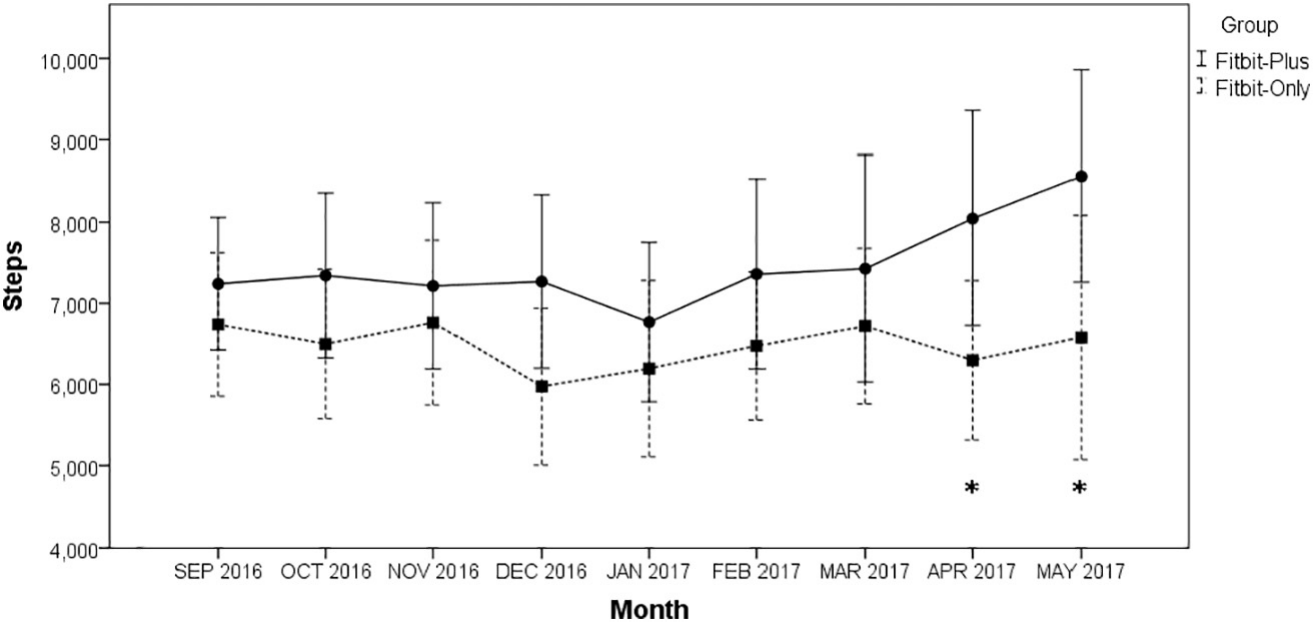
Line graphs for average daily steps of months by groups. *Significant with p-value <0.05.

Daily step data were the primary outcome of this study. As a whole, the mean daily steps were greater in the Fitbit-Plus group than the Fitbit-Only group (7429 ± 2833 vs. 6483 ± 2359), with only months April (8045 ± 3115 vs. 6297 ± 2214, p = 0.028) and May (8561 ± 3001 vs. 6577 ± 2929, p = 0.044) showing significance. For the duration of the 10 months the mean daily steps were significantly greater in women than men in the Fitbit-Plus group, (7248 ± 894 vs. 6619 ± 830, p = 0.011). An interaction between group and gender was significant (p < 0.001). In the Fitbit-Plus group, women (8170 ± 808) showed more daily steps than men (6627 ± 840). In the Fitbit-Only group, however, men showed more daily steps (6611 ± 847) than women (6326 ± 941) (Fig. 2).

Considering the variability of the participants within this study and the fact that ACSM categorizes lean/average body fat % and overweight/obese body fat % by gender, the participants were separated into these categories and further analyzed. The overweight women in the Fitbit-Plus showed a drop (−2.1%) of body fat from baseline, while Fitbit-Only (−0.5%) and Control (−0.9%) showed minimal change. There were no differences found in lean/average men or overweight men for any outcome measures (Table 2. Change in body composition by intervention and by body fat percent categories).

**Table 2:** Change in body composition by intervention and by body fat percent categories.

Baseline, 9 mo		Fitbit-Plus	Fitbit-Only	Control
Weight change, kg				
Lean/average men (6; 7; 7)		0.7 (5.8)	5.9 (8.3)	2.7 (4.6)
Overweight men (9; 11; 12)		4.4 (9.3)	−2.1 (14.2)	0.1 (7.1)
Lean/average women (9; 6; 8)		0.7 (3.7)	−0.5 (5.2)	3.1 (5.0)
Overweight women (10; 12; 13)		−4.0 (3.8)	1.1 (12.7)	−0.2 (6.8)

Body fat change from baseline, %				
Lean/average men (6; 7; 7)		1.1 (1.4)	2.2 (3.8)	2.1 (1.4)
Overweight men (9; 11; 12)		0.3 (2.9)	−1.1 (4.1)	−0.2 (1.9)
Lean/average women (9; 6; 8)		−0.2 (2.4)	−0.5 (1.5)	−0.6 (2.7)
Overweight women (10; 12; 13)		−2.1 (1.6)	−0.5 (4.6)	−0.9 (1.2)

Body mass index				
Lean/average men (6; 7; 7)		<0.1 (0.6)	0.4 (0.5)	−0.3 (0.6)
Overweight men (9; 11; 12)		0.2 (1.7)	−0.1 (2.1)	−0.3 (0.5)
Lean/average women (9; 6; 8)		<0.1 (0.7)	−0.1 (0.5)	−0.1 (0.8)
Overweight women (10; 12; 13)		−0.5 (0.5)	0.4 (2.3)	−0.2 (1.2)

Lean body mass, kg				
Lean/average men (6; 7; 7)		−0.6 (4.7)	1.5 (2.0)	−0.9 (2.8)
Overweight men (9; 11; 12)		2.4 (4.6)	0.9 (5.1)	−6.7 (24.0)
Lean/average women (9; 6; 8)		1.0 (2.4)	0.5 (2.5)	3.1 (5.3)
Overweight women (10; 12; 13)		0.5 (2.1)	1.0 (3.5)	1.3 (4.1)

Fat mass				
Lean/average men (6; 7; 7)		1.4 (2.8)	4.6 (8.2)	4.2 (3.2)
Overweight men (9; 11; 12)		1.8 (7.2)	−3.2 (12.0)	−0.4 (5.8)
Lean/average women (9; 6; 8)		−0.2 (3.6)	−0.9 (3.0)	0.3 (3.2)
Overweight women (10; 12; 13)		−3.8 (3.1)	−1.4 (11.6)	−1.6 (3.2)

Days exercised				
Lean/average men (6; 7; 6)		−0.5 (1.0)	−1.4 (1.0)	<0.1 (2.1)
Overweight men (9; 11; 12)		−0.2 (1.2)	−1.1 (2.2)	−0.6 (2.3)
Lean/average women (9; 6; 8)		0.6 (0.9)	−1.5 (2.0)	−1.0 (1.8)
Overweight women (10; 12; 12)		−0.5 (2.0)	−0.6 (1.2)	−0.3 (1.2)

(N_1_, N_2_, N_3_) represents the sample sizes for the groups of Fitbit-Plus, Fitbit-Only, and Control, respectively.

**Fig. 3:**
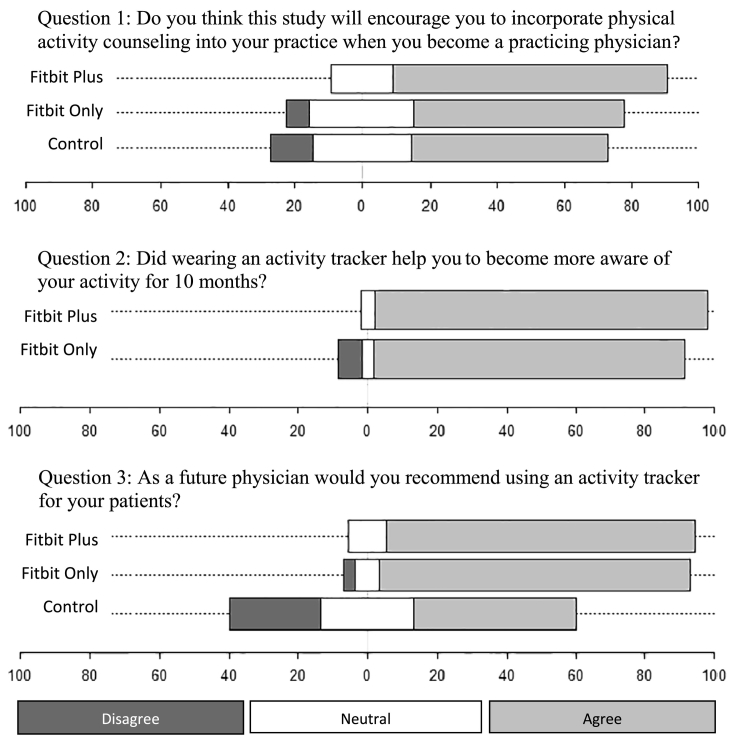
Diverging Stacked Bar Graph for the responses to survey questions.

Participants were asked by survey whether this study would encourage them to incorporate physical activity counseling into their practice when they become practicing physicians. Following the completion of this study, 81.5% of the activity Fitbit-Plus intervention group agreed, 62.1% of just the Fitbit-Only group agreed, and only 58.3% of the Control group agreed (p = 0.25). When asked whether wearing an activity tracker helped them to become more aware of their activity for 10 months, 96.1% of the Fitbit-Plus group agreed and 89.7% of the Fitbit-Only group agreed (p = 0.38). When asked as a future physician if they would recommend using an activity tracker for their patients, 88.9% of the Fitbit-Plus group agreed, 89.7% of the Fitbit-Only strongly agreed, and only 46.7% of the Control group strongly agreed (p < 0.001) (Fig. 3).

## 4 Discussion

The decision to undertake this project with incoming medical students was based on the idea of introducing a future physicians to public health activity recommendations and modern technology to help monitor lifestyle changes. In this study, the addition of weekly emails and group walks combined with an activity tracker was more effective to increase steps compared with an activity tracker alone. Although steps were consistently higher in the Fitbit-Plus group, the only significance noted was months April and May. This may be due to the weather in the geographic region of the study. It is more likely that greater changes will be exhibited better weather during April and May than colder months.

Wearable activity trackers are a popular trend to improve upon physical activity worldwide. Whereas they have been effective as facilitators of activity short term, the long term studies that have been performed show mixed results (Jakicic et al., 2016; Finkelstein et al., 2016; Gualtieri et al., 2016).

Interestingly, the women in the Fitbit-Plus group were consistently higher in step count than the men (8170 ± 808 vs. 6627 ± 840). In the group that were not given any fitness challenges or education (Fitbit-Only) the men had consistently higher steps than the women (6611 ± 847 vs. 6326 ± 941). One rationale for this may be the challenges set forth weekly to the Fitbit-Plus group. When reporting weekly results to this group, the results were reported by separating out the step count by men and women. Although medical schools are approaching 50% enrollment by women, women may feel the need to prove themselves in a field that was once predominantly male. When participants were separated into sub-groups based on body fat percentage, the over-weight women in the group that received fitness challenges had positive results with a decrease in body fat percentage as compared to only wearing an activity tracker or no activity tracker. It is worth noting that lean body mass increased in both women groups (lean/average and overweight). If a person is already at a normal body fat percentage, they have minimal need to change their body fat or to alter body composition. Although the power for the subgroups was low, the descriptive statistics show a positive trend for body fat percent in overweight women and lean body mass in overweight men which warrants further investigation.

The few studies conducted previously that have observed the use of wearable technology at the onset of weight loss programs have been shorter duration studies with small sample sizes and the overall consensus was that utilizing a wearable technology for a duration of <6 months was effective (Coons et al., 2012; Polzien et al., 2007). Jakicic et al. (2016) observed weight loss in overweight participants using a large sample size and examined long term weight loss (24 months). They found that wearable technology did not offer an advantage over standard weight loss approaches (Jakicic et al., 2016).

Body composition is only one of many aspects in improving health. Increased movement and physical activity has been shown to improve biomarkers of health without changes in body composition (Kimata et al., 2018; Smith et al., 2010; Abbott et al., 2004) Increasing physical activity, in particular walking, is correlated with reduced cardiovascular risk factors, increased endurance, increased longevity, improved cognition in older adults and reduced depression rates without any impact on body composition (Kimata et al., 2018; Smith et al., 2010; Abbott et al., 2004). Kimata and colleagues found that by increasing walking distance to just over 1.5 miles a day showed a significant decrease in cardiovascular risk factors compared to older men who walked <1.5 miles daily (Kimata et al., 2018). These studies referenced did not take into account intensity (speed or duration) of walking. These benefits were shown by simply increasing the number of steps. This highlights the importance of this studies results demonstrating the Fitbit Plus group successfully increased step count more than the Fitbit-Only group.

The findings in this study are important for various reasons. The weight of a person, for example, is not an accurate indicator of health. If the results were reported by weight as our outcome measure, the results for this study would not have been positive. Excess adiposity is associated with chronic illness in later life as well as loss of lean muscle mass (sarcopenia) (Cheatham et al., 2018; Maffetone et al., 2017). Improving body composition such as decreased body fat percentage and/or increased lean body mass leads to improved health markers, despite the potential for an increase in weight and/or BMI. It is of public interest for physicians to understand these assessments in order to counsel patients accordingly and not simply use weight or BMI as an indicator of their patient's health status. In the current study there were positive changes in body composition that didn't always lead to decreased body weight. The over-weight men showed no improvement in body fat, however they did increase lean body mass. The results for over-weight women in the Fitbit-Plus group are promising for future clinicians trying to effectively reduce adiposity in women.

There were limitations to this study. The compliance of wearing the activity tracker and syncing their devices was problematic. Twenty-two percent of the participants lost their Fitbits within the study time frame and they had to be replaced. Additionally, there was some bias in our control group with the knowledge that they would have their body composition re-tested following 10 months.

## 5 Conclusions

Utilizing wearable activity trackers with the addition of weekly emails and group walks was more effective to increase activity compared with an activity tracker alone in first year medical students. Additionally, wearing activity trackers with weekly encouragement and feedback may have positive effects on overweight medical students. Further research is warranted to investigate this preliminary finding.
